# Antiretroviral Treatment as Prevention: Impact of the ‘Test and Treat’ Strategy on the Tuberculosis Epidemic

**DOI:** 10.2174/157016211798038524

**Published:** 2011-09

**Authors:** Robin Wood, Stephen D Lawn

**Affiliations:** 1 The Desmond Tutu HIV Centre, Institute for Infectious Disease and Molecular Medicine, University of Cape Town, Cape Town, South Africa;; 2 Department of Medicine, Faculty of Health Sciences, University of Cape Town, Cape Town, South Africa;; 3 Department of Science and Technology/National Research Foundation Centre of Excellence in Epidemiological Modeling and Analysis (SACEMA), University of Stellenbosch, 19 Jonkershoek Road, Stellenbosch, South Africa;; 4 Department of Clinical Research, Faculty of Infectious and Tropical Diseases, London School of Hygiene & Tropical Medicine, London, UK

**Keywords:** Communicable disease control, HAART, highly active antiretroviral therapy, HIV prevention, tuberculosis prevention

## Abstract

Antiretroviral therapy (ART) has been remarkably effective in ameliorating Human Immunodeficiency Virus (HIV)-associated morbidity and mortality. The rapid decline in viral load during ART also presents an opportunity to develop a “treatment as prevention” strategy in order to reduce HIV transmission at a population level. Modelling exercises have demonstrated that for this strategy to be effective, early initiation of ART with high coverage of the HIV-infected population will be required. The HIV epidemic has fueled a resurgence of tuberculosis (TB) particularly in sub-Saharan Africa and widespread early initiation of ART could also impact this epidemic via several mechanisms. The proportion of patients with low CD4 cell counts who are at high risk of TB disease from progression of both latent and new TB infection would be greatly reduced. Entry into a life-long ART program provides an ongoing opportunity for intensified TB case finding among the HIV-infected population. Regular screening for HIV infection also presents an opportunity for intensified TB case finding in the general population. The combined effect of reduced progression of infection to disease and intensified case finding could reduce the overall prevalence of infectious TB, thereby further decreasing TB transmission. In addition, decreasing prevalence of HIV infection would reduce the TB-susceptible pool within the population. The ‘test and treat’ strategy therefore has potential to reduce the TB risk at both an individual and a population level. In this paper we explore the expected “TB dividend” of wider access to ART and also explore the potential of the “test and treat” strategy to impact on TB transmission, particularly in the heavily burdened setting of sub-Saharan Africa.

## INTRODUCTION

The strong association between Human Immunodeficiency Virus (HIV) and increased risk of tuberculosis (TB) was first described in the mid 1980s in the United States (US) [1, 2]. Soon thereafter, the HIV epidemic emerged as an important factor undermining TB control in both industrialized countries and resource-limited settings, especially sub-Saharan Africa [3]. In New York in the early 1990s, the intersection of multi-drug resistant TB and HIV infection resulted in outbreaks of disease in hospitals, prisons and communities, and constituted a serious challenge to public health [4, 5]. However, rigorous implementation of case finding, treatment and prevention interventions in the city quickly brought this rising TB epidemic under control [5]. This was achieved using existing tools and occurred prior to the advent of combination antiretroviral therapy (ART) in the mid-1990s.

In contrast to the focal HIV-associated TB outbreaks and epidemics in the US, which have been amenable to control with traditional TB control interventions, TB has proved to be a far greater challenge in countries with generalized HIV epidemics [6]. In sub-Saharan Africa, HIV has fuelled 3-5-fold increases in TB incidence in many high HIV prevalence countries, especially in the south and east of the continent [6, 7]. In the worst affected countries of South Africa and Swaziland, approximately 1% of the population develops TB each year, a majority of which is HIV-associated. The World Health Organization (WHO) directly observed treatment, short course (DOTS) strategy has failed to control this epidemic even in communities where this has been well implemented and in countries regarded as having model DOTS programmes [7, 8]. It has become clear that DOTS alone is insufficient and that multiple interventions will be required to address the epidemic in sub-Saharan Africa [9].

ART is one of the most potent tools for preventing HIV-associated TB [10, 11] and ART scale-up may therefore play an important role in controlling this epidemic. However, the impact of ART is likely to be highly dependent on how early ART is commenced during HIV progression and on the effective coverage that can be achieved at a population level. Mathematical modelling studies suggest that a strategy of annual HIV testing and immediate initiation of ART for all infected patients regardless of CD4 cell count could have a substantial impact on HIV transmission, thereby leading to reductions in HIV prevalence [12]. However, there will be further benefits of this so-called “test and treat” strategy in reducing the TB risk at both an individual and a population level. In this paper we explore the expected “TB dividend” of wider access to ART and also explore the potential of the “test and treat” strategy to impact on TB transmission particularly in heavily burdened settings in Africa. To better understand this, we first discuss some of the key aspects of the epidemiology of TB before proceeding to describe the impact of ART in cohorts and communities, and we finally consider the potential impact of “test and treat”.

## EPIDEMIOLOGY OF HIV-ASSOCIATED TB

The pathway whereby HIV-infected individuals transition from the TB-susceptible state to TB infection, TB clinical disease and ultimately to TB treatment and cure is outlined in Fig. (**1**). Infection may either progress directly to disease (exogenous TB disease) or enter a period of clinical latency before reactivation at a later stage (endogenous TB disease). Like all other infectious diseases, the primary event determining the disease epidemiology is the acquisition of infection with *Mycobacterium tuberculosis* by a susceptible individual.

### TB Transmission Risk

The incidence of TB infection is very difficult to measure even in high TB burden settings as this requires very large numbers of individuals to be repeatedly tested for infection. Instead, it is usually preferable to measure the prevalence of infection according to age and then mathematically derive the average annual probability of infection [13]. This probability can be described by using two different terms - the current “force of infection” and the mean “annual risk of tuberculosis infection.”

The force of infection is the per capita rate at which susceptible (uninfected) individuals become infected each year and provides an assessment of the current prevailing infection risk in communities with a generalized TB epidemic.

In contrast, the mean annual risk of TB infection (ARTI) is calculated from an observed prevalence of infection at a given age and represents the average annual risk of infection since birth that would produce this prevalence. Thus, force of infection represents current infection risk whereas ARTI is an index of average infection risk over time.

The mean ARTI is the term most often used to describe risk of acquiring TB infection in childhood and has been used to estimate secular trends in transmission within populations [13]. In developed countries such as the United Kingdom (UK), the ARTI has continuously declined from over 10% in 1900, to 1% in the 1950’s and to 0.1% in the 1990’s [14]. However, the ARTI in developing country settings has remained high. In a study of southern African school children, the ARTI was estimated to be between 0.8% and 2.5% in Zambia and between 2.5% and 4.2% in South Africa [15, 16]. In Cape Town, South Africa, the high rate of acquisition of TB infection in African township residents was shown to continue throughout childhood into young adulthood and the force of TB infection was estimated to be 6.8%-7.9% for those between 15 and 20 years of age [17]. A high force of infection results in exogenous primary and secondary TB infections, which will rapidly progress to clinical disease, particularly in HIV-infected individuals with low CD4 cell counts [18].

Fig. (**2**) illustrates the impact of high ARTI and high force of TB infection on patients presenting to an ART clinic in scenarios of low, medium and high TB transmission. Latent infection would be present in approximately 1.3%, 12.4% and 74% in settings where ARTI is 0.04%, 0.4%, or 4% per annum, respectively. Active TB disease in the low and medium transmission settings would be predominantly due to reactivation from latent infection, although limited explosive outbreaks may still occur when the local force of infection is high due to uneven distribution within the population [19].

In contrast, in high transmission settings, the large proportion of individuals with latent infection and the prevailing high force of infection would lead to both increased reactivation and increased primary and secondary TB re-infection disease. Therefore, interventions which modify progression from latent TB infection to active TB disease, such as short-term TB preventive treatment with isoniazid, would be expected to be effective where TB disease was predominantly due reactivation but less so where force of infection is high [20]. In contrast, the relative benefit of ART would be similar in either high or low transmission settings [20].

### Prevalence of Untreated TB

The force of infection is determined by the prevalence of infectious TB (untreated or inadequately treated) in a community and the number of secondary cases resulting from each infectious case (effective contact number). However, the prevalence of TB is also a parameter which is difficult to measure directly and therefore is frequently estimated from other TB parameters such as case notification rates and TB mortality rates [7]. Some data, however, are available. For example, in 2005 prior to widespread access to ART, the prevalence of untreated TB among a population sample of randomly selected individuals in a Cape Town community was 1.6%, with prevalence rates of 5.2% and 0.5% in the HIV-infected and HIV-uninfected populations, respectively [21].

In scenarios where most TB cases eventually enter the TB control program and in a stable epidemic situation, the mean duration of infectiousness before starting effective chemotherapy can be approximated by the untreated TB prevalence divided by the TB incidence rate. Aggressive scale-up of HIV testing and provision of longitudinal HIV care in communities would provide the opportunity for greatly improved TB case finding, thereby reducing the prevalence of untreated TB. This, in turn, would be associated with shortening of the disease duration and reduced TB transmission risk. Thus, to better understand the potential impact of ART on TB transmission within communities, it will be important to assess the impact of ART scale-up on TB prevalence and disease duration.

### Effective Contact Number

The impact of social factors on TB transmission and disease has been long recognised. Social crowding in poorly ventilated housing resulted in exceptionally high TB rates in urbanised populations of the nineteenth century [22]. An effective TB contact is defined as a contact between an infectious pulmonary case and a susceptible individual sufficient to result in TB infection [23]. In the UK, the effective contact number has been estimated to have declined from approximately 22 in 1900 to 10 in 1950 and between 1 and 2 by 1990 [14]. It is assumed that the reduction in effective contact number was a result of steady improvement in social circumstances including decreased household crowding [14].

In Cape Town, the effective contact number is between 11and 22 cases infected for each smear-positive notified case [24]. The effective contact number has epidemiologic importance because if it is less than the reciprocal of the life-time risk of TB disease, the TB epidemic will decline. Thus, for example, when the population life-time risk of developing pulmonary TB is 10%, an effective contact number of less than 10 would be sufficient to result in decline in TB transmission. However, in South Africa with over 5.5 million HIV-infected individuals [25] who have an approximate 10% risk of developing TB each year, both the effective contact number and the prevalence of infectious TB need to be greatly reduced to bring the TB epidemic under control.

## EXISTING TB CONTROL INTERVENTIONS

Combination anti-tuberculosis treatment rapidly decreases the infectiousness of drug-sensitive TB cases [26]. Treatment therefore can effectively decrease ongoing transmission from individuals who are identified by the TB control program and expeditiously started on effective chemotherapy. Thus, the principal TB control intervention of the WHO is the directly observed treatment, short course (DOTS) treatment strategy, and implementation of this has been associated with ongoing improvements in TB control in many settings [7].

The focus of DOTS is on effective case management of self-referred symptomatic patients (passive case finding) and thus removing them from the infective pool. The rates of TB disease are now so low in many industrialised countries that alternative active case finding strategies are no longer considered cost-effective [27]. It has been noted that the annual risk of infection and effective contact number declined markedly in industrialized countries during the first half of the twentieth century even prior to the availability of effective chemotherapy [14]. Thus, the DOTS strategy may have consolidated TB control rather than initiated it.

However, in countries with generalized TB and HIV epidemics, the DOTS strategy has failed to reverse the increasing TB burden. In highly burdened countries, many individuals with HIV-associated TB progress to death without their TB disease being recognised [28-31]. Where HIV is endemic, the effective contact number and rates of progression to active TB are high such that the DOTS strategy may have insufficient impact on the prevalence of HIV-associated TB to reduce ongoing TB transmission. The combination of a high prevalence of unrecognised infectious TB and high effective contact number combine to maintain a high force of infection, which is further amplified by a large number of susceptible HIV-infected individuals, many of whom have advanced immune suppression.

## ANTIRETROVIRALS AND TB RISK

Having described how DOTS is insufficient to address the challenge presented by the HIV-associated TB epidemic in Africa, we now consider the potential role of ART, drawing together data on the burden of TB occurring prior to ART, changing TB risk during in cohorts receiving ART and the modelled and observed impact of ART at a population level.

### Tuberculosis in Patients Initiating ART

South Africa is a country where both HIV and TB are endemic [32]. TB and HIV care services have developed separately although there have been increasing efforts to integrate these programs [33, 34]. The diagnosis of HIV in attendees of TB services has always been a major pathway for access to HIV care and ART provision in South Africa. In a large Cape Town community ART clinic, approximately 16% of referrals between 2002 and 2005 were from the national TB control program [35]. However, following the introduction of provider-initiated HIV testing and counselling within the TB service in 2005, there was a steady increase in the proportion of direct referrals from the TB service to account for 35% of patients in 2007-2008 [35]. The proportion of these patients whose disease was a recurrent TB episode also decreased from 8.6% to 3.2% over the same time period, which is consistent with another report that TB recurrences were approximately halved by ART in Rio de Janeiro [36].

In addition to those HIV-infected patients referred to the ART clinic with a known TB diagnosis, many of the remaining patients accessing the ART service from other referral sources also had TB diagnosed during intensive pre-ART screening (approximately 25%) [37, 38]. Therefore, the total burden of TB disease in those starting ART at this clinic was extremely high. Thus, in the current situation in which patients typically access ART with advanced symptomatic disease, over half the patients at this service have TB at the time of ART initiation, thereby severely limiting the potential of ART as a TB preventive intervention [39].

### Individual Level Impact of ART on TB Risk and TB Mortality

Initiation of ART leads to rapid functional recovery of mycobacterium-specific immune responses, with increasing lymphocyte proliferation of cells which are able to secrete interferon-gamma when stimulated *ex vivo* with mycobacterial antigens [40-43]. Such immunologic responses have been shown to be associated with an enhanced capacity to restrict *in vivo* mycobacterial growth [44]. ART has therefore a potent capacity to improve an individual’s ability to control TB infection and decrease the rate of progression to clinical disease following recent or latent TB infection [44].

In the high burden setting of South Africa (Fig. **3**), use of ART was associated with a TB risk reduction of approximately 80% in patients followed up for 15 months [10]. The reduced TB risk was seen across a broad range of baseline CD4 cell counts and WHO stages of disease [10]. Those patients with advanced clinical stage and with lowest CD4 cell counts had the greatest number of TB cases averted. However, the TB incidence rates remained high over this relatively short period of follow-up at 3.4/100 person-years and 4.6/100 person-years for those with baseline CD4 cell counts <200 cells/mm^3^ and WHO stage 3 or 4 disease, respectively [10].

Data from multiple other cohort studies in both high-income and resource-limited settings report [10, 45-52] reductions in TB incidence of 54-92% in adjusted analyses (Fig. **4A**). A summary analysis of these results [53] estimated an overall risk reduction of 67% (95% confidence intervals [CI] 61-73). Of note, reductions in TB incidence are similar in patients with either positive or negative tuberculin skin tests [54], suggesting that ART impacts the risk of progression to TB disease following either endogenous reactivation or exogenous exposure.

TB incidence rates continue to decline during ongoing ART, as a result of ongoing immune recovery [55-57]. The current CD4 cell count at any given time during ART appears to be the primary determinant of current TB relative incidence risk [55]. A 10-fold reduction in relative TB risk is observed as CD4 cell counts increase from <100 cells/ mm^3^ to >500 cells/ mm^3^ (Fig. **5**). It is still unclear whether incident TB during ART is due to predominantly reactivation or re-infection. However, it has been suggested that in the context of a rising CD4 cell count during ART, reinfection is likely to be the predominant mechanism, whereas both are likely to be important in patients with falling CD4 cell counts [20].

In the Cape Town treatment cohort, the highest TB rates are seen when significant person-time was spent at low CD4 cell counts (<200 cells/ mm^3^); an intermediate risk when CD4 cell counts were in the intermediate range of 200-500 cells/ mm^3^ and the lowest incidence rates when the current CD4 cell counts exceeded 500 cells/ mm^3^ (Fig. **5**) [57]. However, even among these patients with the highest CD4 cell counts, absolute TB rates were still approximately two-fold higher than rates among HIV-uninfected people living in the same community [57, 58].

Case fatality rates are several-fold higher among HIV-infected TB patients than among those without HIV infection, and mortality is related to the degree of immunodeficiency [59, 60]. Analyses of observational cohort data adjusted for baseline patient characteristics (Fig. **4B**) demonstrate that the mortality risk reduction associated with current ART was between 64-95% [61-67]. Further benefits include improved TB treatment outcomes, with some data suggesting an increased rate of sputum smear clearance [61] and a decreased risk of recurrent TB [36].

Current international guidelines recommend ART for all TB co-infected patients regardless of CD4 cell count and it is to be hoped will lead to reductions in TB case fatality rates [68]. However, unfortunately many cases of TB in HIV-infected individuals remain undiagnosed during life and TB remains a significant cause of pre-ART mortality [28-31, 69, 70]. Thus, while the current ART treatment paradigm decreases incident TB and TB case fatality while on ART, the majority of patients present after developing TB disease and so there is little impact on population TB transmission and incidence.

### Modelling Studies of Population Impact of ART on TB

**Fig. (1):**
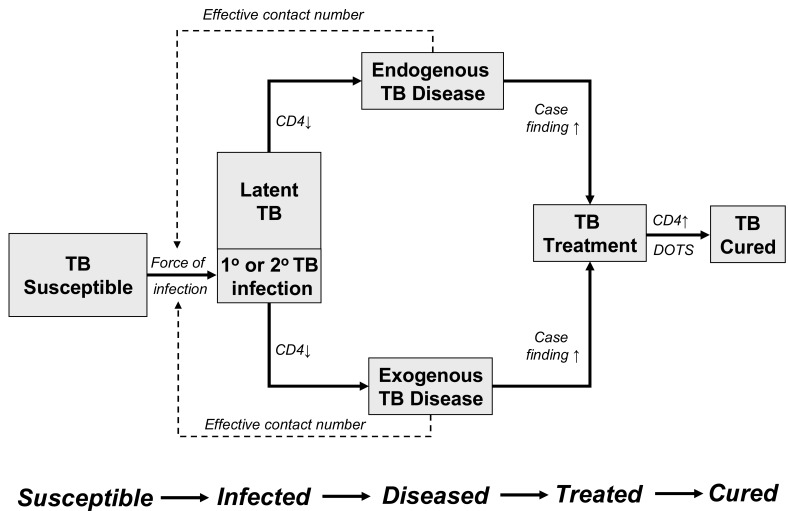
A model for HIV-associated tuberculosis (TB) epidemiology following the pathogenesis of TB from infection, to endogenous or exogenous disease, to treatment and cure. The transition from infection to disease is shown to be CD4 cell dependent. The transition from disease to TB treatment is affected by case finding. The force of TB infection is determined by prevalence of TB disease and the effective contact number. The progression from treatment to cure is primarily affected by the functioning of the directly observed treatment, short course (DOTS) programme.

**Fig. (3):**
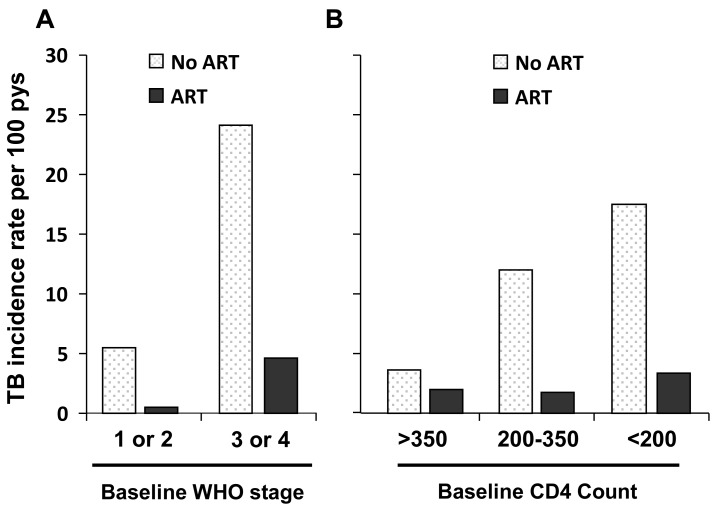
Tuberculosis (TB) incidence (cases per 100 patient years) for HIV-infected patients attending outpatient services in Cape Town, South Africa. Patients were stratified by CD4 cell count and World Health Organization clinical stage. Use of antiretroviral therapy was associated with an approximate 80% reduction of TB incidence over a mean follow-up period of 15 months. (*Data from* Badri M, Wilson D, Wood R. [10]).

**Fig. (4):**
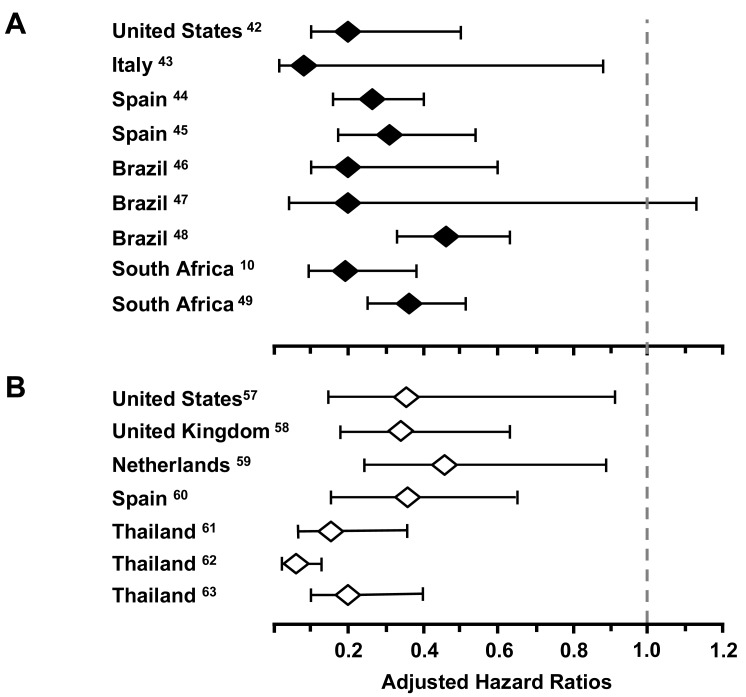
**A**. Adjusted hazard ratios comparing tuberculosis (TB) incidence in HIV-infected individuals receiving antiretroviral therapy (ART) compared with those not receiving ART. Data from nine cohort studies in 5 countries [10, 40-47]. **B**. Adjusted hazard ratios comparing tuberculosis (TB) case fatality rates for HIV-infected individuals receiving antiretroviral therapy (ART) compared with those not receiving ART. Data from seven cohort studies in 5 countries [55-61].

**Fig. (5):**
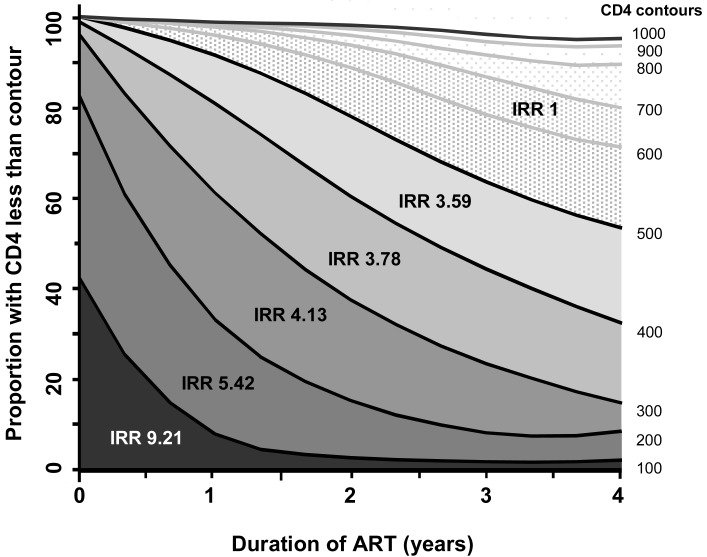
Changes in CD4 cell counts during 4 years of antiretroviral therapy. The graph shows the changes in proportions (%) of patients with CD4 cell counts below the threshold of 1000 cells/ mm^3^ stratified into 100 CD4 cell strata. The tuberculosis (TB) incidence risk ratios (IRR) are shown for each stratum with the comparator being the CD4 cell stratum greater than 500 cell/ mm^3^. The IRR are greatest in the lower CD4 cell strata. The proportions of patients with less than 300 cells/ mm3 decreased over 4 years. (*Data from* Lawn SD, Myer L, Edwards D, Bekker LG, Wood R. [57]).

The impact of ART on TB incidence rates in any given population will depend on its impact on the probabilities of transition from TB susceptibility to TB disease and eventual cure, remission or death (Fig. **1**). In industrialized countries, TB is a rare disease characterised by stochastically-driven small outbreaks rather than by a generalised transmission within the population as a whole [19]. As TB burden has decreased continuously over the last century, the force of infection, latent pool of infection and prevalence of untreated disease are so low that direct measurement would be impracticable.

However, in settings with high TB burdens, knowledge of the size of the different TB-affected populations and impact of ART on the transition probabilities between states is important to predict effectiveness of “test and treat” as a public health intervention. Unfortunately there has been little effort to address the basic epidemiology as there has until recently been a reliance on the DOTS strategy alone. In most heavily burdened settings, the force of infection, latent infected pool, proportion of disease due to recent infection, effective contact number and prevalence of drug-sensitive and drug-resistant infectious TB disease are largely unknown.

HIV-TB epidemic models have been either computer simulations or simple mathematical studies focusing on steady states and their stability. These models generally contain many unknown parameters and rely on few data [71]. Modelling exercises utilising the known individual benefits of ART have been used to try to estimate the population impact of ART. A model incorporating HIV cohort data that included the known rate of CD4 cell decline, the incidence of TB as a function of CD4 cell count, HIV survival and the short-term efficacy of ART in TB prevention identified that early, high ART coverage combined with good compliance would be necessary to substantially impact on TB incidence [72]. It was concluded that the present ART strategies would support curative treatment rather than serve as an effective TB preventive intervention. However, this model did not include impact of ART on either TB transmission or HIV transmission.

Another simple compartmental mathematical model was able to reproduce the historical longitudinal HIV-TB incidence for a single high burdened South African community and indicated that ART could further reduce TB notification rates [71]. This model incorporated the “Styblo Ratio” of a fixed relationship of 10-14 new infections *per annum* for each untreated TB case as a measure of the effective contact number.

Recent mathematical modelling studies have explored the effect of widespread implementation of ART instituted soon after acquisition of HIV infection on the HIV epidemic [12], and these analyses have now been extended to consider the additional impact on the HIV-associated TB epidemic [73]. A model was fitted to the South African HIV epidemic curve and TB notification rates indicated that use of ART as soon as individuals test HIV-positive (the “test and treat” strategy) could rapidly reduce pre-ART TB cases and more slowly impact on-ART incident TB cases. This model incorporated the impact of ART on HIV transmission, with which control of the HIV-associated TB epidemic could be achieved after 4 decades. However, the model assumed no TB transmission interactions between HIV-positive and HIV-negative individuals. Both HIV-positive and HIV-negative adult smear-positive TB index cases have been geographically linked with household acquisition of childhood TB infections [74]. Therefore, reduction in HIV-associated smear-positive disease would be expected to result in some reduction of transmission.

In summary, modelling exercises suggest a probable impact of ART on TB incidence predominantly impacting HIV-infected individuals, with the magnitude of impact dependent on coverage achieved and the time interval between HIV seroconversion and start of ART.

### Observed Population Impact of ART on TB

In contrast to the numerous studies of TB incidence in ART-treated cohorts, there are few data recording the impact of ART on TB incidence or prevalence at a population level. A retrospective analysis of surveillance data from AIDS patients in Rio de Janeiro during the first 3 years of ART provision demonstrated a decline in incidence of TB diagnosed within one year from an AIDS diagnosis from 24.4% to 15.2% [75]. However, another study from Rio de Janeiro covering the same period reported that TB was the primary cause of AIDS deaths, which were often misclassified, and their analysis could not demonstrate any benefit of ART on TB [76].

A prospective observational study was performed in a high HIV and TB burden community of approximately 15,000 persons in South Africa between 2002 and 2008, when access to ART rapidly increased from 0% to 25% of the HIV-infected population [8, 21, 77]. Adult TB notifications increased to a maximum of 2500 cases per 100,000 population between 2002 and 2005, and decreased thereafter to 2000 cases per 100,000 population in 2008 [77].

In observational data, an association between decreasing TB incidence with increasing ART usage does not necessarily prove causality. However, the magnitude of the decrease in TB incidence paralleled the increasing ART use. Moreover, in sub-analyses the decline in TB incidence rate was only observed in the HIV-infected population receiving ART and remained stable in both the other sub-populations of individuals who were either HIV-positive and not receiving ART or who were HIV-negative [77]. The decline in TB incidence is much more likely to have resulted from an improving temporal trend in the CD4 cell count profile rather than from any deterioration in ascertainment of TB among those within the ART program.

The results of two cross-sectional TB prevalence surveys performed in the same South African community in 2005 and 2008 at different stages of ART scale-up are shown in Fig. (**6**) [21, 77]. The TB prevalence among a randomly selected HIV-positive population sample was 9.2 % in 2005 and 3.6% in 2008, while the corresponding ART coverage rates in the community were 13% and 21%. The most marked change between the surveys was a significant (p<0.01) four-fold decrease in laboratory confirmed untreated prevalent cases of HIV-associated TB. This reduction could result either from a decrease in the proportion of individuals with profound immune suppression in the community or from increased TB case finding associated with the implementation of the ART program. It is not possible to distinguish the relative contribution of these two mechanisms.

## THE EFFECTS OF “TEST AND TREAT” ON TB CONTROL

There has been a call to test the efficacy of the “test and treat” strategy in controlled environments [78]. While scale-up of “test and treat” is intended to target HIV incidence and 

long-term HIV prevalence, additional potential impacts on TB epidemic parameters are outlined in Table **1**. These represent the parameters with which the impact of implementation of the “test and treat” strategy might be measured. Such evaluation would require prospective surveillance and detailed and comprehensive surveys within implementing communities.

The primary effects of early and widespread introduction of ART on TB would be by increasing the overall distribution of CD4 cell counts in the HIV-infected population pool. TB risk approximately doubles soon after HIV seroconversion [58] and patients receiving ART and who have CD4 cell counts >500 cells/ mm^3^ have a comparable risk to this [55]. Thus, patients who initiate ART early in the course of HIV infection are likely to remain at approximately just twice the risk of incident TB compared with HIV-negative peers. In contrast, for those who have already progressed to low CD4 cell counts by the time of HIV diagnosis and ART initiation, the time course demonstrated in Fig. (**5**) suggests that it would require greater than 5 years to achieve substantial decreases in the relative risk of incident TB.

Patients receiving ART have greatly improved survival and will remain at increased risk of incident TB. Net reduction in HIV-associated TB rates will be achieved by reduction of time spent at low CD4 cell counts and further impacted by long-term reductions in HIV prevalence. Occurrence of HIV-associated TB at higher CD4 cell counts overall will be associated with lower mortality risk.

The combination of reduced progression from TB infection to TB disease among HIV-infected patients, improved TB case finding among those on ART and incorporation of regular TB symptom screening in the HIV-uninfected population could reduce TB force of infection. Thus, widespread implementation of “test and treat” could use “treatment as prevention” to assist in the control of both the HIV and TB epidemics.

## SUMMARY

There is considerable evidence from observational cohorts that ART improves survival and decreases the rate of progression from both recent and latent TB infection to clinical disease. The population impact of ART is less clear, as increased survival of individuals with low CD4 cell counts could increase the proportion of a population at increased risk of progression to TB disease. In the absence of empiric data, modelling studies have indicated that earlier initiation of ART on a wide scale could significantly impact on TB rates in heavily burdened countries. There is now some early observational data from population studies confirming that TB prevalence and incidence may be reduced by ART.

The population impact of ART on HIV-TB rates may occur by three predominant means. First, ART can improve the CD4 cell count profile of a population and therefore decrease progression from infection to TB disease. Second, HIV-infected individuals on ART receive longitudinal care, providing the opportunity for improved TB case detection and decrease in the prevalence of untreated TB. Third, widespread early implementation of ART may reduce the HIV incidence rate and eventually the prevalence of HIV infection.

Modelling studies have not explored the TB transmission between the HIV-infected and -uninfected population pools as it has been thought that few HIV negatives acquire TB infection from patients with HIV-associated TB. This interaction may have been underestimated because of the long inter-generation time between transmission of infection from HIV-associated TB cases and subsequent development of TB cases in HIV-negative individuals. Further long-term molecular epidemiologic studies will be required to clarify this interaction. Long-term benefits of HIV-TB control for HIV negatives would be an additional impetus for the “test and treat” strategy.

HIV has been considered the primary driver of the explosion of HIV-associated TB epidemic in southern Africa, but it may also be considered to be fuel poured on an existing fire. HIV infection has amplified the high force of TB infection still existing in many parts of the developing world. Control of HIV-associated TB is therefore not isolated from general population TB control. A “test and treat” strategy may provide an additional opportunity for improved general population screening for TB in high burdened settings.

## Figures and Tables

**Fig. (2):**
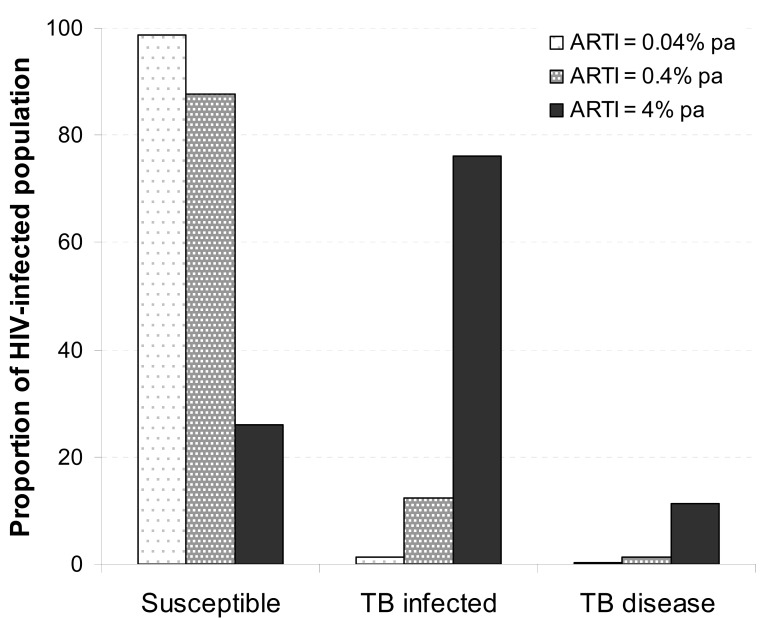
Illustrative values for typical proportions of tuberculosis (TB) infection and disease of HIV-infected patients entering antiretroviral therapy (ART) care in scenarios with low, medium and high TB force of infection. Values of infected proportions are calculated for a cohort with mean age 33 years resident in communities with mean annual risk of TB infection (ARTI) of 0.04%, 0.4% and 4%. (*TB disease data from:* Lawn SD, Fraenzel A, Kranzer K, *et al*. [35]).

**Fig. (6):**
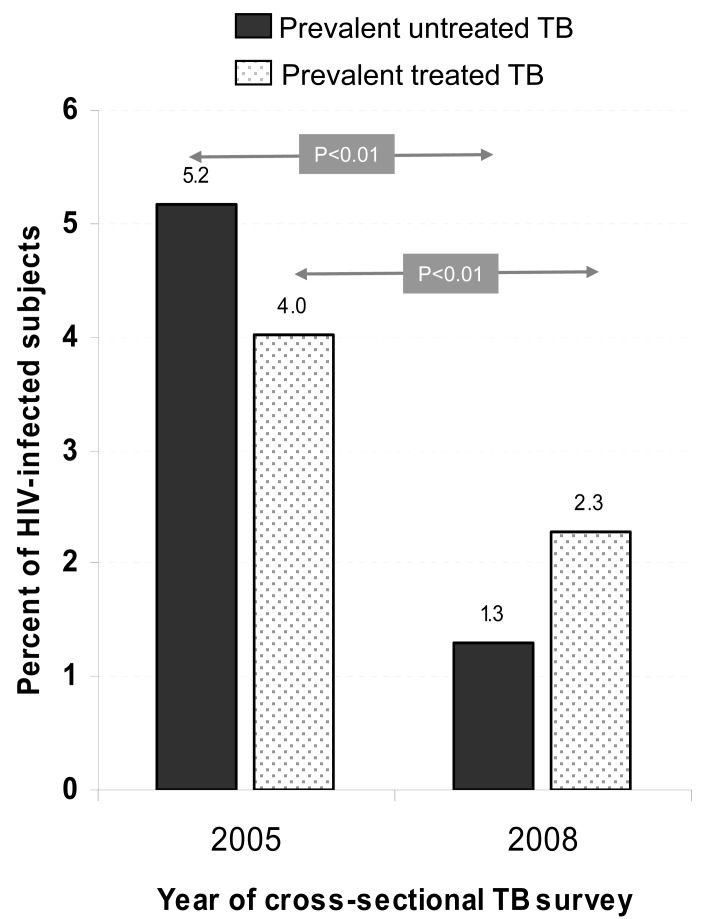
The prevalence of untreated and treated tuberculosis (TB) in random population samples of HIV-infected residents in 2005 and 2008 of a South African township. Untreated TB was confirmed by *Mycobacterium tuberculosis* culture. There was a significant decrease in prevalent TB between the two surveys with the greatest decline in previously unrecognised TB. (*Data from* Wood R, Middelkoop K, Myer L, *et al*. [21] and Middelkoop K, Bekker L-G, Myer L, *et al*. [77]).

**Table 1.** The Potential Additional Impact on the HIV-Associated TB Epidemic of Implementing the ‘Test and Treat’ Strategy of ART Scale-Up Compared to Scale-Up of ART According to Conventional National Plans

	Impact of ‘Test and Treat’ ART Scale-Up
Population CD4 cell count distribution	Increase in median and overall distribution of CD4 cell counts among HIV-infected patients, favourably impacting overall population CD4 cell count distribution
HIV-1 incidence	Reduced by decreasing HIV-1 transmission risk
HIV-1 prevalence	Short-term reductions in prevalence partially offset by increased survival, but substantial long-term reductions
TB case finding	Greatly increased among HIV-infected population during pre-ART screening and ‘unmasking’ of TB during early ART and increased surveillance during ART. Also potential for TB screening to be linked to HIV testing among general population
TB incidence rates in patients receiving ART	Lower due to less cumulative time accrued at low CD4 cell counts
Proportion of HIV-associated TB occurring pre-ART	Greatly reduced with ART initiation at high CD4 cell counts
Proportion of HIV-associated TB occurring during ART	Increased as person-time living with HIV-1 infection accrued prior to ART is greatly reduced.
Total HIV-associated TB rates in the population	Net reduction in HIV-associated TB rates, due to overall reduction in person-time accrued at lower CD4 cell counts and long-term reductions in HIV prevalence
CD4 cell counts among incident HIV-associated TB cases	Much higher median and distribution.
TB prevalence and TB disease duration	Likely to be reduced in HIV-infected population due to improved CD4 count distribution and due to TB screening leading to improved case finding among patients in care
TB transmission	Reduced TB prevalence and disease duration in HIV-infected patients may reduce TB transmission risk
HIV-associated TB mortality	Greatly reduced due to increased case ascertainment and treatment and occurrence of TB at higher CD4 cell counts

TB, tuberculosis; ART, antiretroviral therapy.

## ABBREVIATIONS

ART: = Antiretroviral Therapy
ARTI: = Annual Risk of Tuberculosis Infection
DOTS: = Directly Observed Treatment, Short Course
HAART: = Highly Active Antiretroviral Therapy
HIV: = Human Immunodeficiency Virus
SACEMA: = South African Centre for Epidemiological Modelling and Analysis
TB: = Tuberculosis
UK: = United Kingdom
US(A): = United States (of America)
WHO: = World Health Organization
